# Is Nonmicronized Diosmin 600 mg as Effective as Micronized Diosmin 900 mg plus Hesperidin 100 mg on Chronic Venous Disease Symptoms? Results of a Noninferiority Study

**DOI:** 10.1155/2020/4237204

**Published:** 2020-03-07

**Authors:** Marcio Steinbruch, Carlos Nunes, Romualdo Gama, Renato Kaufman, Gustavo Gama, Mendel Suchmacher Neto, Rafael Nigri, Natasha Cytrynbaum, Lisa Brauer Oliveira, Isabelle Bertaina, François Verrière, Mauro Geller

**Affiliations:** ^1^Hospital Albert Einstein (São Paulo-Brasil), R. Mauricio F Klabin 357/17, Vila Mariana, SP, Brazil 04120-020; ^2^Instituto de Pós-Graduação Médica Carlos Chagas-Fundação Educacional Serra dos Órgãos-UNIFESO (Rio de Janeiro/Teresópolis-Brasil), Av. Alberto Torres 111, Teresópolis, RJ, Brazil 25964-004; ^3^Fundação Educacional Serra dos Órgãos-UNIFESO (Teresópolis-Brasil), Av. Alberto Torres 111, Teresópolis, RJ, Brazil 25964-004; ^4^Faculdade de Ciências Médicas, Universidade Estadual do Rio de Janeiro (UERJ) (Rio de Janeiro-Brazil), Av. N. Sra. De Copacapana, 664/206, Rio de Janeiro, RJ, Brazil 22050-903; ^5^Fundação Educacional Serra dos Órgãos-UNIFESO (Teresópolis-Brasil), Rua Prefeito Sebastião Teixeira 400/504-1, Rio de Janeiro, RJ, Brazil 25953-200; ^6^Instituto de Pós-Graduação Médica Carlos Chagas (Rio de Janeiro-Brazil), R. General Canabarro 68/902, Rio de Janeiro, RJ, Brazil 20271-200; ^7^Department of Medicine, Rutgers New Jersey Medical School-USA, 185 S Orange Ave., Newark, NJ 07103, USA; ^8^Hospital Universitário Pedro Ernesto, Universidade Estadual do Rio de Janeiro (UERJ) (Rio de Janeiro-Brazil), R. Hilário de Gouveia, 87/801, Rio de Janeiro, RJ, Brazil 22040-020; ^9^Universidade Federal do Rio de Janeiro (UFRJ) (Rio de Janeiro-Brazil), Av. das Americas, 411/1613-2, Rio de Janeiro, RJ, Brazil 22631-000; ^10^Laboratoire Innotech International, 22 Avenue Aristide Briand, 94110 Arcueil, France

## Abstract

**Background:**

Phlebotonics have beneficial effects on some symptoms related to chronic venous disease (CVD) of the lower limbs. The most commonly used one is diosmin, available in a pure semisynthetic form or as a micronized purified flavonoid fraction. *Patients and Methods*. The primary objective of this single-blind, randomized, parallel-group, prospective study was to assess the clinical noninferiority of nonmicronized diosmin 600 mg once daily (D-group) compared to micronized diosmin 900 mg plus hesperidin 100 mg once daily (D/H-group) over a 6-month treatment period. Adult patients with a symptomatic CVD of the lower limbs (C0-C3 grade; 20-60 mm on a 100 mm visual analog scale (VAS)) were included. The primary endpoint was the change (from baseline to last postbaseline value) of the intensity of the lower-limb symptoms on VAS.

**Results:**

114 patients (mean age, 44.4 years; women, 90.4%) were randomized in the per-protocol analysis (D-group, *n* = 57; D/H-group, *n* = 57; D/H-group, *p* < 0.0001) in the D-group and -22.8 mm (*p* < 0.0001) in the D-group and -22.8 mm (*p* < 0.0001) in the D-group and -22.8 mm (

**Conclusion:**

Nonmicronized diosmin 600 mg was proven to have a noninferior efficacy compared to micronized diosmin 900 mg plus hesperidin 100 mg, associated with greater ease in swallowing the tablet.

## 1. Introduction

Chronic venous disease (CVD) refers to a broad range of abnormal clinical changes arising from morphological and functional abnormalities of the lower extremity venous system, especially the incompetence of superficial, deep, and perforating veins [1]. “Chronic venous insufficiency” is often mistakenly used as a synonym of CVD, but it should be restricted to the latest stages of the disease [2]. The disease is characterized by an increase of venous pressure in the lower limbs and subsequent inflammatory and trophic changes of the skin and subcutaneous tissues, especially in the most severe stages [3]. The symptoms and signs of CVD attributed to inflammation and pressure on adjacent nerves by dilated veins include pain, sensation of swelling, heaviness sensation, and leg tightness [3, 4]. These symptoms are chronic and progressive and they can alter significantly the quality of life of patients [5]. In industrialized countries, the prevalence of the disease is 2–6.4/1000 with an increased frequency in women and elder individuals [1].

Treatments used in CVD are aimed at improving functional symptoms and preventing complications, and they are classified into two categories: invasive (e.g., sclerotherapy and surgery) or conservative (e.g., elastic compressive bandage, drugs, and local treatment) [6]. Due to their ease of administration, oral treatments are frequently proposed to patients [7]. Phlebotonics are a heterogenous therapeutic class; most of them are natural flavonoids extracted from plants or semisynthetic compounds with flavonoid properties [8]. These treatments are associated with beneficial effects on both macrocirculation and microcirculation generally by improving venous tone and by decreasing capillary hyperpermeability [9, 10]. A recent Cochrane review analysed 53 trials reporting randomized clinical trials with phlebotonics (mainly rutosides, diosmin, hidrosmin, and calcium dobesilate) in CVD [11]. The authors concluded that moderate-quality evidence supported the beneficial effects of phlebotonics on edema and on other signs and symptoms (e.g., trophic disorders, cramps, restless legs, swelling, and paresthesia) when compared to placebo; nonetheless, there was no difference with placebo in ulcers which are a late consequence of the chronic venous disease [11].

Diosmin is one of the most used phlebotonics worldwide. The diosmin-containing medicinal products available on the market contain either pure semisynthetic nonmicronized diosmin or micronized diosmin. Semisynthetic nonmicronized 600 mg diosmin demonstrated a tonic effect on veins, a protective effect on vessels, and anti-inflammatory effects [12, 13]. Micronization allows improving the intestinal absorption of drugs. However, previous exploratory studies reported comparable functional improvement of symptoms after one month of treatment with one 600 mg tablet of pure diosmin versus two tablets of 450 mg diosmin and 50 mg hesperidin as micronized purified flavonoid fraction (MPFF) [14–16].

Micronized diosmin 900 mg plus hesperidin 100 mg is now available as a single tablet. This formulation was recently launched on the market to allow one daily intake instead of two. According to the Marketing Authorization Holder (MAH) of the medicinal products containing MPFF, the combination of purified flavonoid fraction (containing diosmin and hesperidin) and micronization allows increasing the clinical efficacy compared to pure and nonmicronized diosmin. In this trial, we tested the hypothesis that, despite different expected bioavailability and slightly different active ingredients (hesperidin differs from diosmin only by a double bond), there was no impact in terms of clinical efficacy on venous symptoms. This noninferiority trial is the first to compare diosmin 600 mg to MPFF 1000 mg, both administered as a single tablet per day over a 6-month treatment period.

## 2. Patients and Methods

### 2.1. Study Design

This noninferiority, single-blind, randomized, and parallel-group prospective study was performed in six Brazilian university centers from June 2017 to March 2018.

The primary objective of the study was to demonstrate the clinical noninferiority of nonmicronized diosmin 600 mg tablets compared to micronized diosmin 900 mg plus hesperidin 100 mg tablets in adult patients with symptomatic CVD after 6 months of treatment. The secondary objectives were oral acceptability of the study treatment, global satisfaction of the patient, global satisfaction of the physician, and safety.

Written informed consent was obtained from each patient. The protocol was conducted in accordance with the Declaration of Helsinki and Good Clinical Practices and was approved by local independent Ethics Committees (Centro Universitário Serra dos Órgãos (UNIFESO)—approval no. 1.941.780). This study is registered with the ClinicalTrials.gov identifier NCT03471910.

### 2.2. Patient Population

Patients were included if they met the following criteria: patients of both genders > 18 years old; presenting C0 to C3 venous disease grade of the lower limbs, according to the CEAP classification [14]; and clinical symptoms (heavy legs, painful legs, tired legs, sensations of swelling, and/or tension in the legs) of chronic venous disease of the lower limbs as defined by a 100 mm VAS rated by the patient between 20 and 60 mm on the most symptomatic leg.

The main exclusion criteria were as follows: treatment by compression stocking within the 2 months before inclusion; treatment by phlebotonics within the 2 months before inclusion; known allergy or hypersensitivity to any component of the study drug; known clinically significant laboratory alterations; CEAP levels 4–6; patient with venous disease requiring surgery or chemical endovenous ablation; patient suffering from a painful pathology other than venous pain in the lower limbs; patient with a history of venous thrombosis or thromboembolic disease within 6 months before inclusion; and alteration of general condition incompatible with participation in the trial. Women who were pregnant, breastfeeding, or of child-bearing potential not using acceptable birth control methods for the duration of the study were also ineligible.

### 2.3. Study Procedures

Randomization to study drug groups was generated using a random-allocation software. Randomization was performed sequentially for two groups, in blocks of 4, with a 1 : 1 ratio between the treatment groups.

After inclusion visit, visits were scheduled at months 2, 4, and 6. In addition to physical examination and vital sign measurement, venous symptoms were rated at each visit by the patient using a 100 mm visual analog scale (VAS) (from 0 = ^“^absence of venous symptoms^”^ to 100 = ^“^maximal intensity of venous symptoms^”^). This VAS globally assessed the venous symptomatology of the most symptomatic leg (heavy, painful, tired leg, sensation of swelling, or tension). The difficulty to swallow the study medication was also rated by the patient using a 100 mm VAS (from 0 = ^“^very easy to swallow^”^ to 100 = ^“^very difficult to swallow^”^). The global satisfaction of patients and investigators related to the treatment efficacy was assessed using a 4-level scale (bad, acceptable, good, and very good). Tolerability was assessed by the record of adverse events and compliance to treatment by the treatment units returned by the patients.

The study was blinded for patients, but the investigators could identify the study drug (size of tablets). There was a unique packaging for the treatment units with no label of the allocated study drug.

Treatment compliance was calculated based on the number of tablets (repackaged in boxes of 66 tablets) provided to each subject and the number of tablets returned at each study visit as follows: compliance = 100 × ((66 − number of tablets returned)/duration). The compliance was capped at 100% to avoid overestimation.

### 2.4. Study Drugs

According to randomization, patients of the D-group received nonmicronized diosmin 600 mg (one coated tablet, once daily in the morning, Flebodia®, MAH: Laboratoire Innotech International) and patients of the D/H-group received micronized diosmin 900 mg plus hesperidin 100 mg (one coated tablet, once daily in the morning, Daflon®, MAH: Laboratórios Servier do Brasil Ltda.). The 6-month duration of treatment for both groups was chosen according to the minimal duration of treatment in the Summary of Product Characteristics of the investigated product containing diosmin plus hesperidin in Brazil. Some treatments or practices were prohibited during the study: phlebotonic drugs, food or dietary supplements with claimed phlebotonic effect, compression stocking of more than 10 mm Hg, and participation in another clinical trial.

### 2.5. Sample Size

The sample size calculation was based on the change in intensity of the lower limb symptoms (VAS symptom score) from baseline to last postbaseline value. With a noninferiority bound set at 20 mm, the statistical hypotheses were null hypothesis, *M*_D_–*M*_D/H_ ≥ 20 mm, and alternate hypothesis, *M*_D_–*M*_D/H_ < 20 mm, with *M*_D_ as the mean VAS change in the D-group and *M*_D/H_ as the mean VAS change in the D/H-group. If the null hypothesis was rejected, the clinical noninferiority of diosmin 600 mg was demonstrated.

With a noninferiority margin fixed at 20 mm and a standard error estimated at 30 mm, the number of subjects required was 39 in each group to have 90% power to test noninferiority with a one-sided 0.05 significance level. With an expected rate of 35% of major deviations (due to a high expected rate of dropouts), the total number of patients to be enrolled was 120.

### 2.6. Statistical Analysis

The statistical analysis plan was approved and signed before the clinical database lock and treatment unblinding for the study. Three analysis populations were defined: the intent-to-treat (ITT) population included all randomized patients who received at least one dose of treatment, the per-protocol (PP) population included the patients from the ITT population with no major protocol deviations, and the safety population included all patients who received at least one dose of treatment. Less than 4 months of treatment was defined as a major protocol deviation. The per-protocol population was the primary population for efficacy assessment as recommended [15, 16].

Baseline characteristics were described and compared between treatments using a two-sided *t*-test for continuous variables (nonparametric Wilcoxon test if the normality of the data was not verified) and a chi-square test or Fisher's exact test for categorical variables.

The primary endpoint was analysed by a covariance analysis with the treatment as the factor and the baseline VAS symptom score as the covariate. The interaction treatment × covariate was tested but removed from the model if not significant. With a noninferiority bound estimated at 20 mm, noninferiority was declared if the one-sided 95% upper confidence limit of the difference *M*_D_–*M*_D/H_ computed by the covariance model was less than 20 mm.

Continuous secondary endpoints, such as the intensity of the lower limb symptoms at each time and the difficulty to swallow (both measured on a 0-to-100 VAS), were analysed by a mixed model with repeated measures with the treatment and the visit as fixed factors, the subject as a random factor, and the value at baseline as a covariate. The interaction treatment × visit was tested. Treatment estimates and differences were deducted from the model at each visit.

Patient satisfaction and investigator satisfaction were analysed using a generalized linear random model for multinomial data with treatment and time as fixed factors and the subject as a random factor. The interaction treatment × visit was tested and intertreatment contrasts at each visit were evaluated.

Tests were two sided and the significance level was 0.05. Normality was tested by the Shapiro-Wilk test at the 1% threshold.

Safety and compliance data were displayed descriptively.

## 3. Results

### 3.1. Disposition of Patients

**Figure 1:**
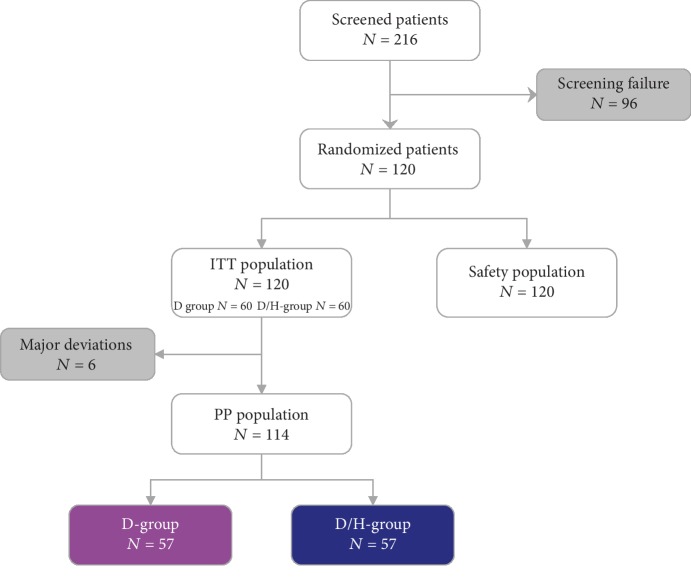
Flow chart.

A total of 216 patients were screened in six centers and 96 patients not meeting the inclusion criteria were excluded. One hundred and twenty patients were randomized: 60 patients in the D-group and 60 patients in the D/H-group (ITT population and safety population) (Figure 1). Early study discontinuations occurred for three patients in the D-group and four patients in the D/H-group. Six out of the seven discontinuations occurred after patients had received less than four months of treatment and were considered as major deviations. Therefore, the per-protocol population was composed of 57 patients in the D-group and 57 patients in the D/H-group.

### 3.2. Baseline Characteristics of Patients

Patients of the per-protocol population had a mean age of 44.4 years and a mean body mass index (BMI) of 25.9 kg/m^2^, and 90.4% were women (Table 1). Severity of chronic venous disease was rated C1–C3 according to the CEAP classification for 99.1% of patients. On 100 mm VAS, the mean intensity of venous symptoms was rated with a mean of 48.7 mm by patients. Demographic and clinical data were comparable at baseline except the intensity of venous symptoms (47.1 mm on VAS for the D-group and 50.3 mm for the D/H-group; *p* = 0.03, Mann-Whitney test). There was also a slight between-group difference for the reference leg: right leg for 40.4% of patients in the D-group and 57.9% of patients in the D/H-group (*p* = 0.061).

### 3.3. Efficacy of Study Drugs on Symptoms of Chronic Venous Disease

In the per-protocol analysis, venous symptoms improved from baseline to endpoint in both study groups: the observed mean change was -23.8 mm in the D-group and -23.9 mm in the D/H-group (Table 2). In order to take into account the baseline VAS, a covariance analysis was performed with the treatment as the studied effect and the baseline VAS as the covariate. There was no interaction baseline × treatment (*p* = 0.38). The estimate-adjusted mean changes were -24.9 mm (*p* < 0.0001) in the D-group and -22.8 mm (*p* < 0.0001) in the D/H-group. The upper limit bound of the 90% confidence interval (CI) of the adjusted difference between the D-group and the D/H-group was +1.0 mm. As the noninferiority bound was fixed at 20 mm, noninferiority (upper limit of 90% CI < noninferiority bound) was demonstrated at the 5% significance level. This result means that there were 95% chances that the VAS improvement from baseline with 600 mg nonmicronized diosmin (D-group) was, at worst, 1 mm lower than the VAS improvement from baseline obtained with micronized diosmin 900 mg plus hesperidin 100 mg.

The noninferiority was confirmed in ITT analysis with the upper limit bound of the 90% CI of the adjusted difference between the D-group and the D/H-group that achieved +1.5 mm.

**Figure 2:**
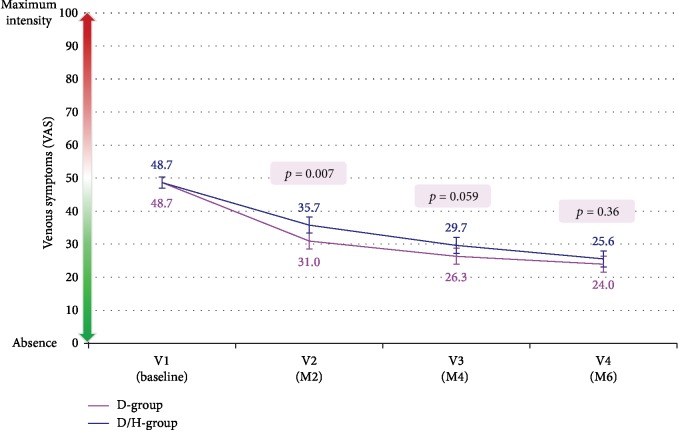
Time course of venous symptoms during the study assessed with a visual analog scale (results are given as adjusted means ± SE).

The intensity of venous symptoms (VAS) significantly decreased at month 2 in the D-group compared to the D/H-group for adjusted means (standard error (SE)): 31.0 (1.2) vs. 35.7 (1.2) mm (*p* = 0.007), respectively (Figure 2). At month 4, venous symptoms were also less intense in the D-group compared to the D/H-group, but statistical significance was not achieved: 26.3 (1.2) vs. 29.7 (1.2) mm (*p* = 0.059). At month 6, the two groups were comparable for venous symptoms.

### 3.4. Ability to Swallow the Study Drug

**Figure 3:**
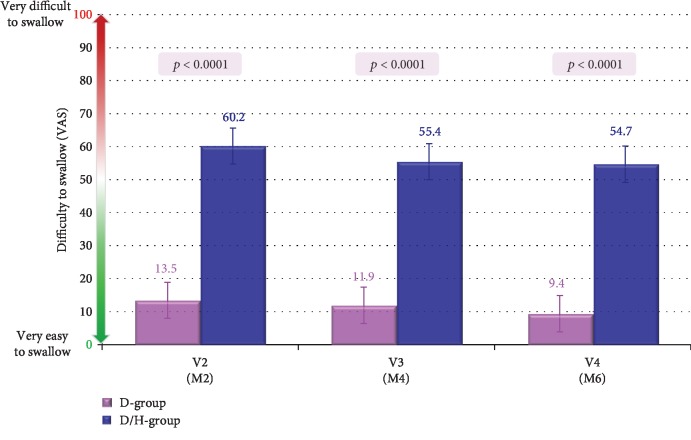
Difficulty to swallow assessed with VAS (results are given as adjusted means ± SE).

The difficulty to swallow the tablets of the study drugs was assessed using a VAS. The study drug was significantly easier to swallow in the D-group compared to the D/H-group at all visits (Figure 3). Thus, at month 6, adjusted means (SE) of VAS was 9.4 mm in the D-group and 54.7 mm in the D/H-group (*p* < 0.0001).

### 3.5. Global Satisfaction of Investigators and Patients

The global satisfaction of the investigators was comparable for the two study drugs. Overall, satisfaction was rated as good-very good during the study by a large majority of investigators: 79.0% in the D-group and 76.8% in the D/H-group at month 6 (*p* = 0.55).

The global satisfaction of the patients was better in the D-group compared to the D/H-group at month 2: satisfaction was rated as good-very good by 77.2% of patients in the D-group and 50.9% in the D/H-group (*p* = 0.04). For the other visits at months 4 and 6, patients' global satisfaction was comparable in both groups.

### 3.6. Safety

No serious adverse event was reported. One severe adverse event (diarrhea) was reported in the D-group. At least one adverse event possibly or probably related to study drugs was reported in 21 (35.0%) patients of the D-group and 16 (26.7%) patients of the D/H-group. The most frequent (>5%) adverse events related to treatment were nausea (13.3% and 20.0% in the D-group and the D/H-group, respectively), dyspepsia (16.7% and 6.7%, respectively), diarrhea (8.3% and 5.0%, respectively), headache (6.7% and 6.7%, respectively), and vertigo (6.7% and 3.3%, respectively).

There were two patients in the D-group and one patient in the D/H-group with at least one adverse event that led to treatment discontinuation. These adverse events were palpitations (*n* = 1), abdominal pain (*n* = 1), diarrhea (*n* = 1), and dyspepsia (*n* = 1) in the D-group and vertigo (*n* = 1) and weight decrease (*n* = 1) in the D/H-group (more than one adverse event could be reported per patient).

### 3.7. Compliance to Treatment

Median compliance in the ITT/safety population was 98.4% (interquartile range, 96.8-100) at each visit. Only two patients showed compliance < 80% at month 2 (they were excluded from the per-protocol population). Median global compliance was 98.9% in both groups for ITT and per-protocol populations.

## 4. Discussion

The population included in our study was mainly composed of women (90.4%), and patients were relatively young with a mean age of 44.4 years and a BMI of 25.9 kg/m^2^. According to the inclusion criteria, patients were rated C0-C3 (CEAP classification) with a good balance between classes, meaning that they had no skin trophic disorders and no ulcers. On a 100 mm VAS, patients evaluated the baseline symptom intensity at 48.7 mm.

At 6 months, the clinical noninferiority of nonmicronized diosmin 600 mg compared to micronized diosmin 900 mg plus hesperidin 100 mg was demonstrated. During the 6-month follow-up, the intensity of the symptoms steadily decreased and VAS values at month 6 were approximately half of the baseline values. In the D-group, the mean decrease of symptoms was significantly more pronounced at month 2 than in the D/H-group. This difference was, however, not clinically significant, and for the next visits, the mean intensity of symptoms progressively met the mean value of the D/H-group.

This finding is in accordance with those of previous studies comparing the therapeutic efficacy of daily doses of nonmicronized diosmin 600 mg versus micronized diosmin 900 mg and hesperidin 100 mg. Improvements in CVD symptoms, self-assessed by patients using VAS, were generally rapid and significant compared to those in the baseline in both treatment groups after treatment [14, 15, 17]. However, these studies were exploratory in design; treatment duration was generally shorter (28 days); and dosages, patient population, disease severity, number of tablets (2 tablets 500 mg MPFF), or galenic formulations were often different, which was not enough for a definite conclusion on the relative effectiveness of both diosmin formulations.

The better ability to swallow the study drug reported in the D-group compared to the D/H-group is likely related to the large size of the tablet of micronized diosmin 900 mg plus hesperidin 100 mg which should not be broken, opened, or chewed according to the manufacturer. In daily clinical practice, dissolution of the tablet in a glass of water is, however, possible, but with the risk of underdosing. Drug acceptability is a challenge in long-term treatment of chronic diseases such as CVD [18]. The ease in swallowing an oral medication is an important component of drug acceptability, and any strategy that enhances acceptability improves the adherence to drug treatment [19].

The global satisfaction of the investigator was comparable in both study groups with a high degree during the entire follow-up. There was nevertheless a trend for a lower investigator global satisfaction at month 2 in the D/H-group compared to the D-group; at the same visit, the global satisfaction of the patient was significantly lower in the D/H-group (good-very good for 50.9% of patients vs. 77.2% in the D-group; *p* = 0.04). This lower degree of satisfaction at the first visit after treatment initiation could be related to the lower swallowability reported in the D/H-group. One notes also that a significantly lower efficacy was reported in the D/H-group compared to the D-group at the same visit. It could be suggested that a lower compliance could explain a lower efficacy of the study drug due to the lower oral acceptability. However, the data of treatment compliance do not support this hypothesis because compliance remained high throughout the study including at the 2-month visit. Finally, we cannot exclude that the difficulty to swallow had a negative impact on the perception of symptoms. Indeed, there is an affective dimension of pain, particularly in patients with chronic pain, which could be modulated by a negative or positive mood [20].

Safety was good and no serious adverse event was observed in both groups, which is consistent with the known safety profiles of both tested drugs. The chemical structures of diosmin and hesperidin are very similar. At the dosing tested, the expected difference in terms of systemic exposure to diosmin between the micronized diosmin-containing product and the pure diosmin-containing product did not translate into a difference in terms of safety. Only one severe adverse event (diarrhea) was reported in the D-group. According to the Summary of Product Characteristics of the pure nonmicronized diosmin investigated product (Flebodia®), the possibility of minor digestive troubles leading rarely to stoppage of the treatment is reported. The Summary of Product Characteristics of the micronized diosmin plus hesperidin investigated product (Daflon®) reports also minor digestive troubles as common adverse events. In the large French study of Cazaubon et al. which included 1442 patients who received 600 mg of nonmicronized diosmin once a day (drinkable suspension or tablet), only 22 patients had adverse events with a relationship to study drug not excluded (digestive disorders); 6 patients (0.4%) discontinued the study [21]. The higher rate of adverse events reported in our study could be related to the 6-month duration of treatment (versus one month in the study of Cazaubon et al.).

The strong points of this study are mainly the head-to-head comparative design, the allocation concealment, the 6-month study duration, the large number of patients, and their assessment in the context of a noninferiority design. Indeed, assessing noninferiority is very demanding in terms of patient follow-up and adhesion to protocol. Only six patients out of 120 had major protocol deviations, and per-protocol and ITT analyses were concordant, thus ensuring the robustness of the conclusions. In addition, the primary endpoint was a VAS which, as described in the Cochrane analysis of Martinez-Zapata et al. [11], has been used in many trials to assess the efficacy of phlebotonics on CVD symptoms [17, 22–29].

However, although well conducted, this study has some limitations. It was blind for the patients but not for the investigators. Performing a double-blind trial would have required two placebo tablets for each patient with the same size and aspect than the verum. In this case, each patient would have swallowed two tablets (including a big one) and acceptability could not have been compared. The bias related to the absence of double blinding was probably limited since the primary endpoint was assessed by patients who were not aware of the name of the medicinal products. Another limitation of the study is the absence of a placebo arm. It may be acceptable for a noninferiority study when previous trials already demonstrated that the reference product has a large amplitude effect compared to placebo. With regard to phlebotonics, it is recognized that the placebo effect accounts for a significant part of their efficacy in CVD [30]. However, the aim of the present study was not to establish the “specific” effect of a well-established phlebotonic drug but to compare two medications for their overall clinical effect and by taking also into account their acceptability.

## 5. Conclusion

In conclusion, the clinical noninferiority of nonmicronized diosmin 600 mg compared to micronized diosmin 900 mg plus hesperidin 100 mg was demonstrated with an acceptability in favor of nonmicronized diosmin 600 mg. The duration of the study (6 months) met the minimal duration of treatment recommended in Brazil for the micronized diosmin 900 mg plus hesperidin 100 mg investigated product in a population of patients with CVD. These results suggest that a unique dose of pure diosmin 600 mg and not micronized is not less efficient than a dose of 900 mg of micronized diosmin which is supposed to make diosmin much more bioavailable and efficient. In addition, the medication intake was easier for the tablet of diosmin 600 mg alone, which may likely improve treatment adherence that is challenging in CVD as in other chronic conditions. Additional investigations in larger patient populations may be required to confirm the present results and to clarify the relationship between the daily dose of diosmin, the absorbed amounts of active diosmin metabolites, and the patients' perception of clinical outcomes.

## Figures and Tables

**Table 1:** Characteristics of patients at baseline (per-protocol population).

Characteristics	D-group *N* = 57	D/H-group *N* = 57	Total *N* = 114
Age (years), mean (SD)	43.2 (11.2)	45.6 (10.3)	44.4 (10.8)
Female gender, *n* (%)	52 (91.2)	51 (89.5)	103 (90.4)
Ethnicity, *n* (%)			
Asian	1 (1.8)	0	1 (0.9)
Caucasian	32 (56.1)	27 (47.4)	59 (51.8)
Black	9 (15.8)	9 (15.8)	18 (15.8)
Brown	15 (26.3)	21 (36.8)	36 (31.6)
Body mass index (kg/m^2^), mean (SD)	25.7 (3.4)	26.0 (3.6)	25.9 (3.5)
Reference leg, *n* (%)			
Right	23 (40.4)	33 (57.9)	56 (49.1)
Left	34 (59.6)	24 (42.1)	58 (50.9)
CEAP classification, *n* (%)			
C0^a^	0	1 (1.8)	1 (0.9)
C1	21 (36.8)	20 (35.1)	41 (36.0)
C2	25 (43.9)	21 (36.8)	46 (40.4)
C3	11 (19.3)	15 (26.3)	26 (22.8)
Medical history, *n* (%)			
Ear, nose, and throat	7 (12.3)	3 (5.3)	10 (8.8)
Cardiopulmonary	7 (12.3)	10 (17.5)	17 (14.9)
Digestive system	10 (17.5)	6 (10.5)	16 (14.0)
Nervous system	4 (7.0)	7 (12.3)	11 (9.6)
Musculoskeletal system	3 (5.3)	3 (5.3)	6 (5.3)
Skin	7 (12.3)	6 (10.5)	13 (11.4)
Others	3 (5.3)	8 (14.0)	11 (9.6)
Venous symptoms (VAS) (mm)	47.1 (8.2)	50.3 (9.5)	48.7 (9.0)

VAS: visual analog scale; S: symptomatic. ^a^The patient rated C0 was symptomatic.

**Table 2:** Clinical efficacy of diosmin 600 mg once daily (D-group) compared to diosmin 900 mg plus hesperidin 100 mg once daily (D/H-group) in noninferiority analysis (per-protocol population).

	D-group *N* = 57	D/H-group *N* = 57
Venous symptoms (VAS) (mm)		
Baseline	47.1 (8.2)	50.3 (9.5)
Endpoint	23.3 (8.6)	26.4 (11.7)
Change endpoint-baseline	-23.8 (10.8)	-23.9 (12.6)
Percentage of variation	-49.6 (19.4)	-47.0 (23.0)
Adjusted means of the changes (least square means)		
Estimate-adjusted mean (SE) baseline	-24.9 (1.3)	-22.8 (1.3)
*t*-value	-18.7	-17.1
Pr > | *t*|	<0.0001	<0.0001
90% CI	-27.1; -22.7	-25.0; -20.6
Difference D − group − D/H − group (least square means)		
Estimate difference (SE)	-2.1 (1.9)	
*t*-value	-1.12	
Pr > | *t*|	0.2648	
90% CI	-5.3; 1.0	

Pr: probability; SE: standard error; VAS: visual analog scale.

## Data Availability

Derived data supporting the findings of this study are available from the corresponding author (MG) on request.
